# Supplementary material for: Discovery of Physics From Data: Universal Laws and Discrepancies
Source: Front Artif Intell. 2020 Apr 28;3:25. doi: 10.3389/frai.2020.00025 (PMC7861345; doi:10.3389/frai.2020.00025)
Supplement: Supplementary file 1 [file Data_Sheet_1.PDF]

Supplementary Material

1 SPARSE IDENTIFICATION OF NONLINEAR DYNAMICAL SYSTEMS

In this section we provide some additional information concerning the Sparse Identification of Nonlinear Dynamical systems (SINDy) method. We first give a simplified implementation of the sequentially thresholded least-squares algorithm, implemented in Python, before showing examples of SINDy applied to two test problems: a nonlinear oscillator (Section 1.1) and a simulated falling body with different types of drag (Section 1.2).

Recall that to construct a set of governing equations, SINDy seeks to solve the following optimization problem

$$\min_{\boldsymbol{\Xi}} \frac{1}{2}\left\|\dot{\mathbf{X}} - \boldsymbol{\Phi}(\mathbf{X})\boldsymbol{\Xi}\right\|_F^2 + \Omega(\boldsymbol{\Xi}), \tag{S1}$$

where $\mathbf{X}$ is a matrix of measurements, $\dot{\mathbf{X}}$ is a matrix of derivatives of $\mathbf{X}$, $\boldsymbol{\Phi}(\mathbf{X})$ is a library matrix whose columns consist of potential right-hand side functions evaluated on the measurement data, $\boldsymbol{\Xi}$ is a coefficient matrix, and $\Omega(\cdot)$ is a regularization term encouraging sparsity. A one-dimensional version of the sequentially thresholded least-squares algorithm, which we use to solve (S1) in this work[1], can be implemented in Python as

```python
xi = least_squares(theta, x_dot)  # Initial guess

# delta is our sparsity parameter
for k in range(iterations):
    small_indices = abs(xi) < delta
    big_indices = ~small_indices

    xi[small_indices] = 0  # Threshold small coefficients
    xi[big_indices] = least_squares(x[:, big_indices], x_dot)
```

Here we use `least_squares` to denote a black-box least-squares solver. This implementation only solves for one column of the coefficients of $\boldsymbol{\Xi}$, yielding the governing equation for only one measurement variable. In practice one runs this routine for each variable.

1.1 A brief example

Here we give an example of a dynamical system SINDy is easily able to identify: a first order nonlinear oscillator. The system is described by

$$\begin{aligned} \dot{x} &= -\frac{1}{10}x^3 + 2y^3 \\ \dot{y} &= -2x^3 - \frac{1}{10}y^3. \end{aligned} \tag{S2}$$

To construct training data for a SINDy model we simulate a trajectory under these dynamics starting from $(2, 0)$ for $t \in [0, 5]$ with a time step of 0.01. Using a threshold of 0.05 and a library consisting of

[1] In actuality we use a custom implementation of sequentially thresholded least-squares for the main paper and most of the supplementary material and a recently developed package, PySINDy (`https://github.com/dynamicslab/pysindy`), for the examples in this section.

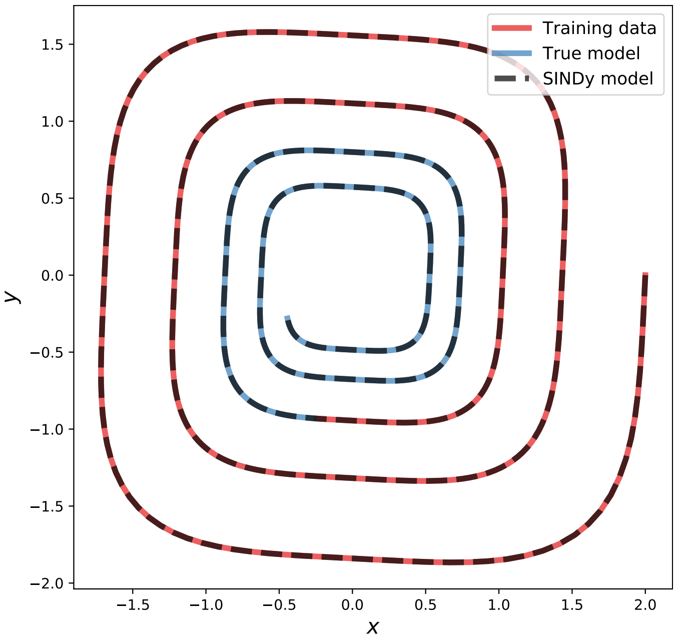

Figure S1. Dynamics of the nonlinear oscillator described by (S2). The true trajectory, computed using (S2) is plotted as a solid line, with red denoting the training data fed to the SINDy model and blue denoting the portion of the trajectory unseen by SINDy. The dashed line shows the dynamics predicted by the model discovered by the SINDy model starting at initial condition $(2, 0)$.

polynomials terms of degree up to five, SINDy recovers the following model

$$\dot{x} = -0.100x^3 + 1.999y^3$$
$$\dot{y} = -1.999x^3 - 0.100y^3.$$

We plot the trajectories simulated from the actual model and the SINDy model for $t \in [0, 25]$ in Figure S1. Note the close agreement between the two trajectories.

1.2 Learning equations of motion

In this section we demonstrate that SINDy can readily learn simplified versions of the equations of motion, but struggles to identify dynamics containing terms not representable as linear combinations of the library terms. We simulate a ball of unit mass falling with constant acceleration and no drag

$$\dot{v} = -9.8, \qquad v(0) = 0$$

and with constant acceleration and linear drag

$$\dot{v} = -9.8 - 0.5v \qquad v(0) = 0.$$

Each simulation consists of 50 height measurements taken every fifteenth of a second. We numerically differentiate the height data, then feed the velocity profiles to SINDy models with thresholds of 0.1. SINDy

learns the following governing equations:

$$\dot{v} = -9.8000 \qquad \text{(drag-free simulation)},$$
$$\dot{v} = -9.786 - 0.499v \qquad \text{(linear drag simulation)}.$$

Multiple factors contribute to the accuracy of the learned models for these two test cases. The effects of the constant acceleration and drag on the ball trajectories are relatively large, the data lacks noise, and the appropriate terms are present in the trial libraries used by the SINDy models.

On the other hand, when a higher fidelity drag model is used—one which contains terms missing from and poorly approximated by the library functions—SINDy struggles to identify coherent dynamics. Using the drag model given in (S3) and (S4) (see Section 4) to simulate a falling ball, SINDy learns the governing equation

$$\dot{v} = -6.345$$

for a "large" threshold value (0.1). The constant acceleration term is shifted away from the true value to compensate for the drag. For a small "threshold" (0.004), SINDy learns the following model

$$\dot{v} = -9.810 - 0.005v + 0.17v^2.$$

The constant acceleration is very close to the true value, but there is also a nonphysical positive quadratic term. Without including rational and other more complicated nonlinear functions in the library[2], SINDy lacks the proper building blocks to perfectly reconstruct the behavior of the system. Poor performance can be a signal that some information is not being captured by the library, which is typically chosen based on one's underlying assumptions about the dynamics being studied. In this way SINDy can help reveal discrepancies between the assumed form of the governing equations and reality without necessarily exposing the precise nature of the discrepancy. If one finds that SINDy is producing unreliable models, a possible remedy is to enrich the library of candidate right-hand side functions.

2 NUMERICAL DIFFERENTIATION

In this section we explore the error introduced by smoothing and numerical differentiation. More specifically in Section 2.1 we compare the performance of a few methods of numerical differentiation, in Section 2.2 we examine the effects of smoothing on noisy data, and in Section 2.3 we approximate the level of noise present in the actual ball drop data set and use the results of the previous sections to derive estimates for the error in the numerical derivatives used in the paper.

Unless otherwise noted, we worked with a single synthetic trajectory consisting of height measurements generated from an idealized falling object obeying

$$\dot{v} = -9.8 - 0.5v, \qquad v(0) = 0, \qquad x(0) = 40.$$

This particular model was chosen because it is qualitatively similar to the actual trajectories. The measurements are taken at a rate of 15 per second to further imitate the experimental setup. We then add various amounts of Gaussian noise to the measurements. In the plots that follow "noise level" refers to the

[2] Including rational functions in the library introduces additional complications to the SINDy algorithm Mangan et al. [2016].

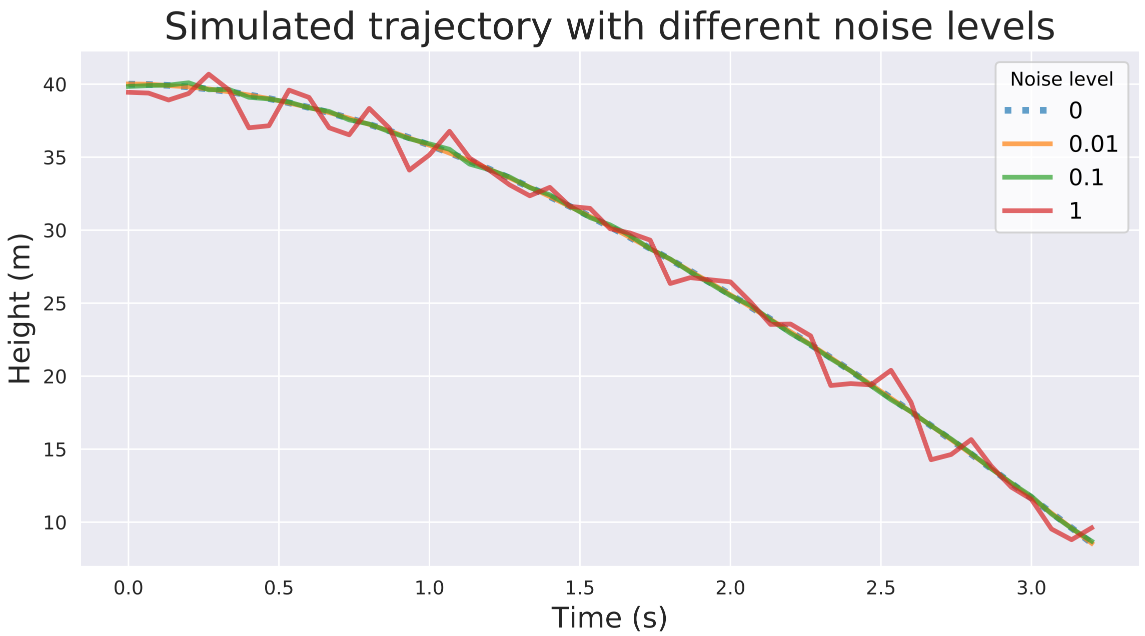

Figure S2. The simulated trajectory used for our numerical differentiation and smoothing experiments with varying amounts of noise added.

standard deviation of the noise added. Figure S2 shows the trajectory with various amounts of noise. Note that even a noise level of 0.1 is almost indistinguishable from the true trajectory.

2.1 Differentiation method comparison

We evaluate four numerical differentiation variants: two (first order) forward difference methods and two (second order) centered difference methods. For one method of each order we apply Savitzgy-Golay smoothing before performing computing the derivative. For the remaining two methods (one first order and one second order) we do not smooth the data before taking the derivative. We use a window size of 35 when performing smoothing. We compute both the first derivative (velocity) and second derivative (acceleration) of the simulated trajectory since the associated differential equation is second order.

Figure S3 summarizes our results. There are a few observations to be made:

- The smoothed versions of the methods exhibit much better performance than the unsmoothed variants as the noise level increases.
- Once enough noise is introduced, all the methods considered see their accuracy degraded roughly linearly with the noise level.
- The error levels are higher for the approximate acceleration than for the velocity. This makes sense since some error is introduced in computing the velocity and the velocity is needed to compute the acceleration.
- At a low enough noise level there tends to be little difference between the smoothed and unsmoothed versions of each method. The unsmoothed centered difference method outperforms its smoothed counterpart in computing the velocity of relatively clean data.

A conclusion we can draw from this analysis is that the smoothed centered difference method provides the best performance over most levels of noise for both the first and second derivatives.

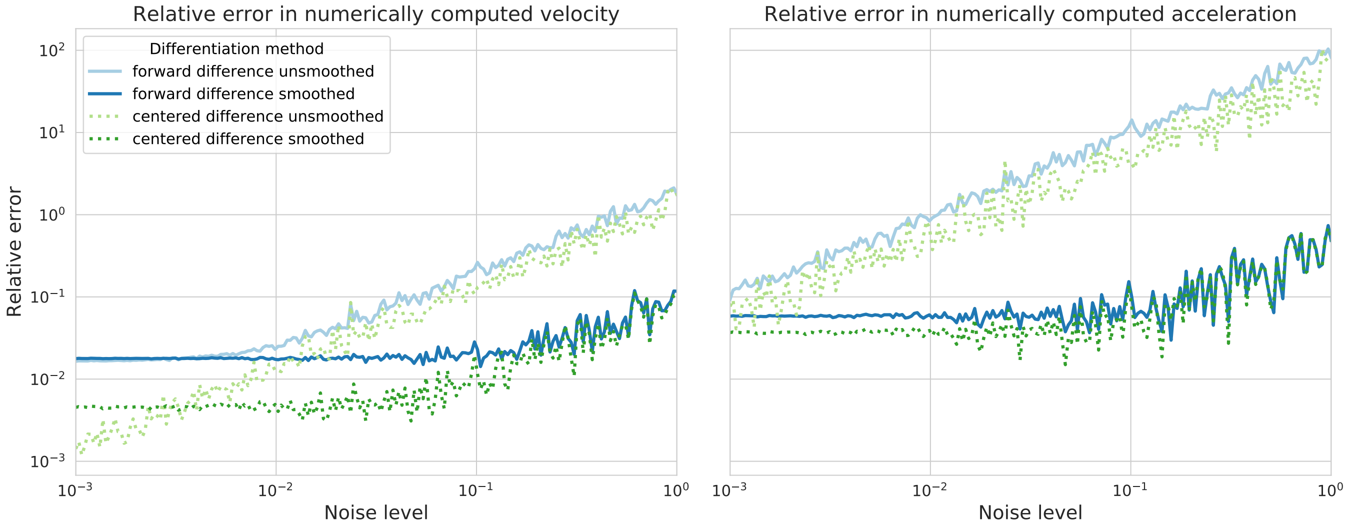

Figure S3. Left: Relative error in the *first* derivative of the trajectory computed using four differentiation methods with varying amounts of noise. Right: Relative error in the *second* derivative of the trajectory computed using four differentiation methods with varying amounts of noise.

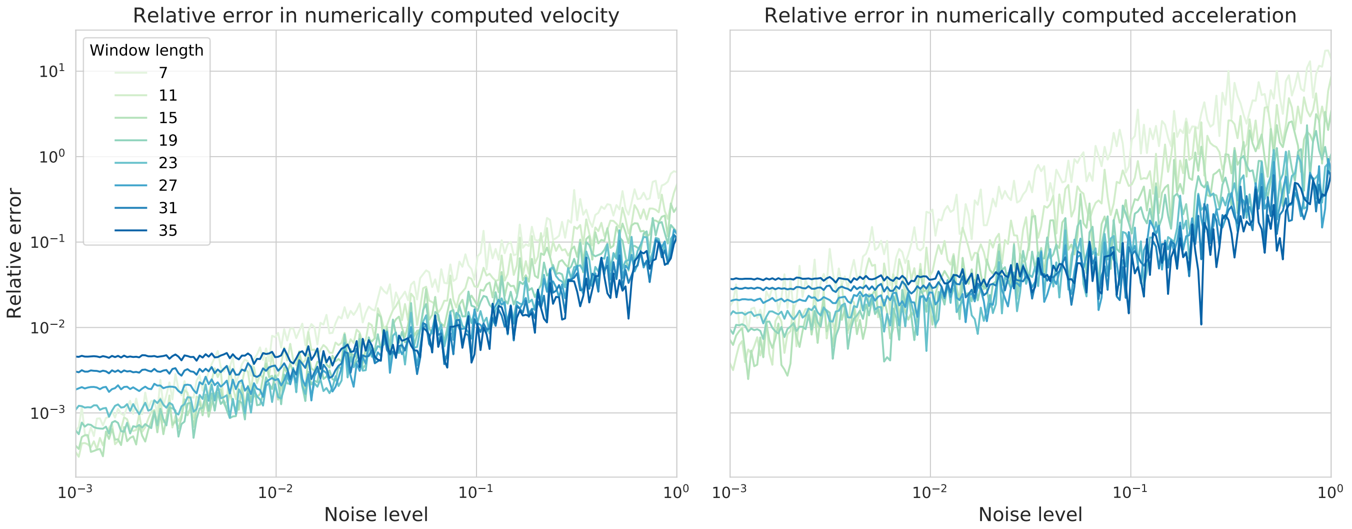

Figure S4. The effects of the size of the smoothing window on derivative approximation error. Left: Relative error in the *first* derivative of the trajectory computed using a smoothed centered difference method with different smoothing window sizes. Right: Relative error in the *second* derivative of the trajectory computed using a smoothed centered difference method with different smoothing window sizes.

2.2 Smoothing

In the previous experiment we used a fixed window length without justifying our choice. In this section we fix the differentiation method used — centered difference with smoothing — and vary the window length. A larger window means that more points are considered when performing smoothing. The window length roughly translates to smoothness; the larger the window the smoother the result.

Figure S4 plots how the error in numerically computed derivatives is affected by the size of the smoothing window used as a function of noise. For small noise levels, larger smoothing windows hurt the method; overly aggressive smoothing throws out some useful information. As the noise levels increase the opposite

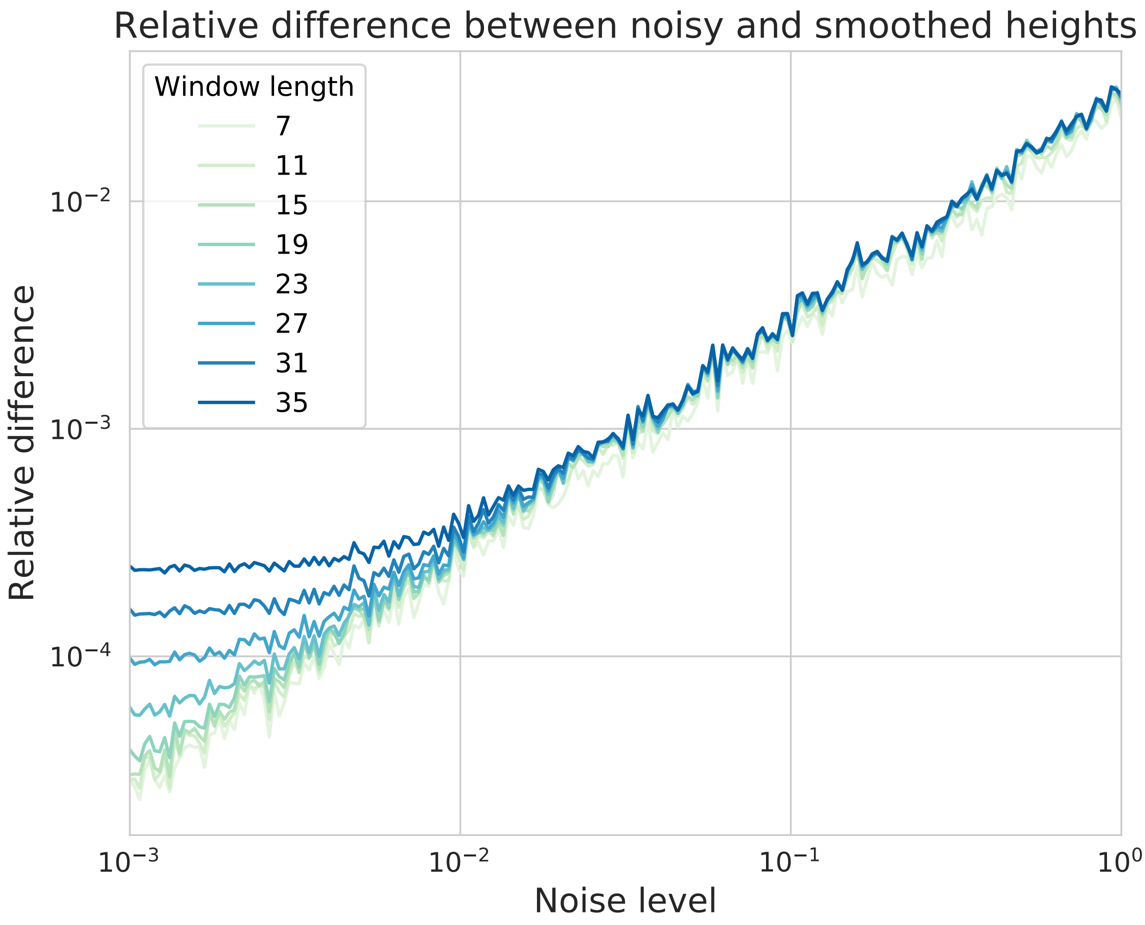

Figure S5. The relative difference between noisy trajectories and their smoothed versions for different length smoothing windows.

is true; larger amounts of smoothing are needed to keep the excessive noise at bay. Which window length we should actually use will depend on the noise level we suspect is present in the real-world data set.

2.3 Estimating noise in measurement data

In order to infer the amount of noise in the measured ball trajectories it will prove useful to know roughly how much the act of smoothing a trajectory changes the underlying data. To this end we perform a similar experiment as in the previous section, but with the height data itself. That is to say we apply the same smoothing operation used before to the height data and measure the relative difference between the smoothed and original data. For completeness, we carry out this experiment for multiple window lengths.

Our results are shown in Figure S5. As one would expect, smaller windows produce smoothed trajectories that are closer to their unsmoothed counterparts, but only slightly so. Large smoothing windows have the most pronounced effects when the noise levels are very small and smoothing is unnecessary. For higher noise levels, changes in window size affect the relative difference very little.

Based on these results we elect to use the largest window size tested in our experiments in the main paper. SINDy depends heavily on accurate numerical derivatives. For large amounts of noise, a larger window size is necessary for numerical differentiation to work well. We are only penalized for using a large window (in the sense that we greatly modify the original data when we perform smoothing) if the underlying noise level is below about 10^{-2}.

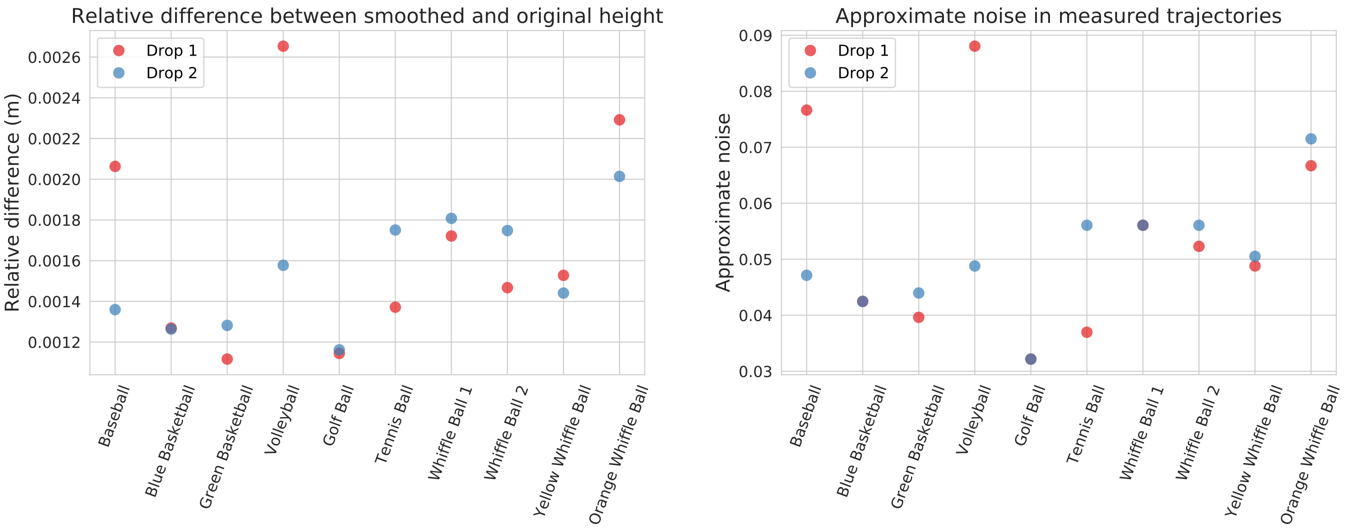

Figure S6. Left: Relative difference between the smoothed and unsmoothed falling ball trajectories for both drops (window length = 35). Right: Approximate noise levels present in the ball drop measurements.

Next we turn to the task of actually estimating the noise present in the drop data. To accomplish this we apply smoothing with a window length of 35 to each falling ball trajectory, then measure the relative difference between the smoothed and unsmoothed versions. Finally we compare this relative difference with Figure S5 to obtain an approximation to the noise level. Figure S6 visualizes the relative differences along with the inferred noise levels for each ball drop. The estimated noise levels are all between 0.035 and 0.065. Comparing these results with Figure S3, we can deduce that the numerically computed velocity and acceleration vectors have relative errors of order 10^{-3} and 10^{-2}, respectively. It should be noted that we use the ℓ^2 norm when computing relative error. If the ℓ^∞ norm is used instead, the relative errors increase marginally.

3 EFFECT OF VARYING SPARSITY PARAMETER

In this section we provide a representative example of the that are produced when the sparsity threshold parameter is varied. We consider both the regularized and unregularized SINDy variants. The effect of varying the sparsity parameter is similar In both cases. For large values of the parameter (reflecting a strong preference toward a very sparse solution), no terms are deemed "important" enough to be retained and the trivial model is returned. As the threshold is continuously decreased, a small number of terms will be selected for a range of threshold values. Eventually, when the threshold becomes small enough, suddenly there will be a noticeable jump in the number of terms in the models returned by SINDy. This is typically when one can assume that the sparsity parameter has been made too small. We demonstrate this pattern in Tables S1 and S2, which give the learned equations for unregularized and unregularized SINDy, respectively, for a variety of sparsity thresholds. The parameter values were chosen to be close to values at which the number of terms in the resulting models changed. For the experiments carried out in the paper we chose thresholds which were slightly larger than the values at which the jumps in numbers of model terms occurred.

In cases where one wishes to perform automatic parameter tuning, cross-validation should allow for one to choose an appropriate sparsity parameter value. Models that are overly sparse (those which have too large a sparsity parameter) will be too simple to accurately predict unseen data and models that are

Threshold	Equation
10	$v' = 0$
2	$v' = -7.6344$
0.1	$v' = -14.865 + 0.1084x - 0.2914v$
0.005	$v' = -6.1068 - 0.0717x + 0.0880v - 0.0059xv$
0.0045	$v' = -2.9116 - 0.1388x + 0.0861v - 0.0061xv - 0.0048v^2$
0.0035	$v' = 14.7998 - 0.6964x + 0.7036v - 0.0182xv + 0.0039x^2 - 0.0065v^2$
0.002	$v' = 45.4998 - 1.4749x + 2.4829v - 0.0559xv + 0.0067x^2 - 0.0393v^2 - 0.0021v^3$

Table S1. Models learned by unregularized SINDy for different threshold parameters (tennis ball, drop one).

Threshold	Equation
70	$v' = 0$
65	$v' = -6.9$
2	$v' = -8.3 - 0.1v$
0.2	$v' = -15.6 + 0.1x - 0.3v$
0.14	$v' = -2.0 - 0.1x + 0.4v - 0.01xv$
0.1	$v' = 1.5 - 0.2x + 0.4v - 0.01xv + 0.001v^2$
0.05	$v' = -13.1 + 0.2x - 0.6v + 0.008xv - 0.003x^2 - 0.009v^2$
0.02	$v' = 21.7 - 0.7x + 1.7v - 0.04xv + 0.002x^2 - 0.05v^2 - 0.003v^3$
0.01	$v' = -35.2 + 1.1x - 4.7v + 0.13xv - 0.01x^2 - 0.2v^2 - 0.0009x^2v + 0.003xv^2 - 0.001v^3$
0.005	$v' = 9.9 - 0.8x - 0.8v - 0.04xv + 0.02x^2 - 0.005v^2 + 0.0004x^2v - 0.0004xv^2 - 0.0002x^3 - 0.0003v^3$

Table S2. Models learned by regularized SINDy for different threshold parameters (all balls, drop one).

not sparse enough (those which have too small a sparsity parameter) will overfit the training data and will generalize poorly. Poor performance on the holdout/validation/test set should catch both overfit and underfit models.

One troublesome case that is possible with SINDy is when the model jumps directly from underfitting to overfitting as the sparsity parameter is varied. This could occur for a number of reasons, but the primary suspects are typically:

- The library is not rich enough to properly capture the dynamics (i.e. one or more of the terms in the “true” underlying dynamical system are not present in the library being used by SINDy).
- The library is too rich. If too many functions are included in the library then the system solved by SINDy can become ill-conditioned, leading to unpredictable results.
- The data are not described by a dynamical system. If this is the case then SINDy is not an appropriate tool.
- Data are too noisy. Recovering a sparse solution from extremely noisy data may not be possible.

4 REALISTIC FALLING BALL SIMULATIONS

In the main work we simulate falling balls with *constant* drag proportional to the balls’ velocities. However, in reality, the drag varies nonlinearly with Reynolds number, which it itself a function of velocity. In this section we discuss what the two SINDy models are able to learn when a more complicated, but physically accurate model is used to construct the synthetic ball drops. Specifically, to simulate falling spheres, we numerically solve the following initial value problem for 49 time steps of length $1/15$ seconds (to mimic

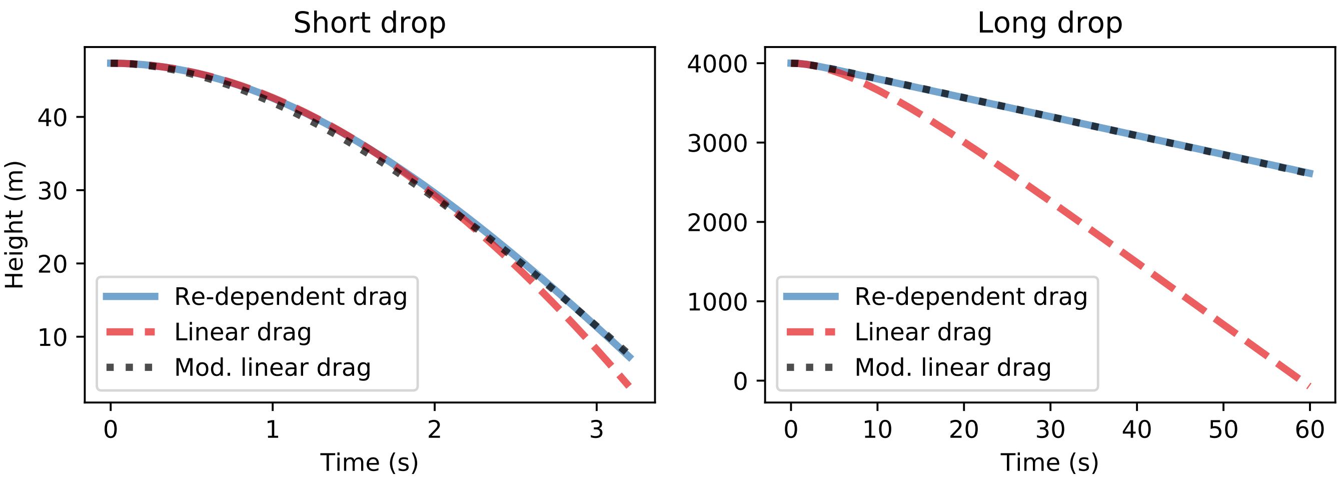

Figure S7. A comparison of the simulated trajectories of a tennis ball using a Reynolds number-dependent drag force from (S3) and (S4) (solid) and two different constant linear drags (dashed and dotted). On the left we show a short drop similar to the physical experiments and on the right we have simulated a longer drop lasting a full 60 seconds. For the Reynolds number-dependent drag model we used the mass and diameter of the actual tennis ball. For the first linear drag model we used constant gravitational acceleration and a drag coefficient of -0.125 (the average of the two drag coefficients selected by SINDy in the real-world experiments). The modified linear drag model involved constant acceleration of $-12.7\ \mathrm{m/s^2}$ and a drag coefficient of -0.53. No noise was added.

the real-world experiments)

$$m\dot{v} = mg + \frac{1}{2}\rho v^2 A C_D, \qquad v(0) = 0 \tag{S3}$$

where m is the mass of the ball, ρ is the density of air, $A = \pi r^2$ is the cross-sectional area of the sphere, r is the radius of the sphere, and C_D is the Reynolds number dependent drag coefficient. We use $\rho = 1.211\ \mathrm{kg/m^3}$ (the density of air at sea level with a temperature of 65 degrees Fahrenheit). For C_D we use the following approximation which is based on experimental measurements, recommended in Brown and Lawler [2003]:

$$\frac{24}{Re}\left(1 + 0.150 Re^{0.681}\right) + \frac{0.407}{1 + \frac{8710}{Re}}. \tag{S4}$$

This approximation is valid for $Re < 2 \times 10^5$, just before the so-called "drag crisis" when C_D drops suddenly. We do not attempt to reproduce the behavior of the drag coefficient during and after the drag crisis, only before it. This model also assumes that the spheres are smooth. In Figure S7 we compare the simulated trajectories for a tennis ball using both the drag force from (S3) and (S4) and the linear drag model presented in the main paper. Note that the trajectories initially agree, but as the ball reaches higher velocities and Reynolds numbers, the more complicated Re-dependent model predicts a larger drag force. The difference between these two models becomes clear when the simulations are run for longer amounts of time (900 time steps). The ball effected by linear drag reaches a much faster terminal velocity compared with the other ball. Figure S7 also shows how a linear drag model with a larger drag coefficient (and larger constant acceleration) can mimic the Re-dependent model.

As before we simulate five idealized balls falling for 49 time steps of duration $1/15$ seconds, each with a different mass and radius. The masses and radii were selected to match a subset of the balls in the real-world data set. Table S3 gives the characteristics of each simulated ball. Varying amounts of noise are then added

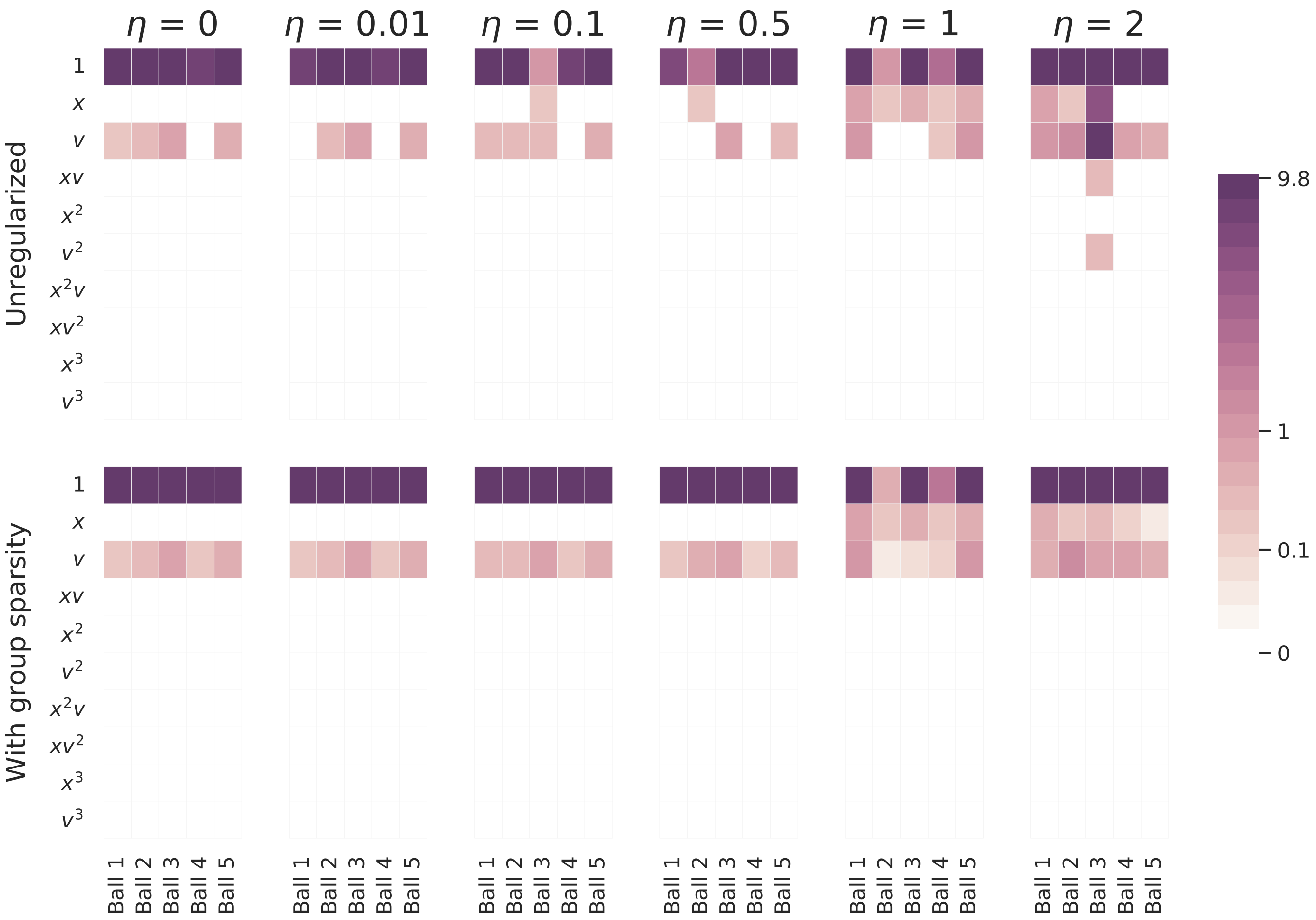

Figure S8. A comparison of coefficients of the models inferred from the simulated falling balls. The top row shows the coefficients learned with the standard SINDy algorithm and the bottom row the coefficients learned with the group sparsity method. η indicates the amount of noise added to the simulated ball drops. The standard approach used a sparsity parameter of 0.05 and the group sparsity method used a value of 0.3. The balls' trajectories were simulated using equation (S3).

to the artificial measurement data. Finally, we apply the unregularized and group variants of SINDy. The coefficients learned by the two methods are shown in Figure S8.

Simulated ball	Real ball	Radius (m)	Mass (kg)
Ball 1	Golf Ball	0.022	0.0454
Ball 2	Tennis Ball	0.033	0.0567
Ball 3	Whiffle Ball 1	0.036	0.0283
Ball 4	Baseball	0.035	0.1417
Ball 5	Blue Basketball	0.119	0.5103

Table S3. Properties of the simulated balls and the real balls after which they were modeled.

The unregularized SINDy models exhibit better performance here than in the linear drag case in the sense that they tend not to pick up extraneous terms such as x until relatively high noise levels are present. Notably, many of the models include constant acceleration and linear drag terms. The group sparsity methods perform similarly as before. For low noise levels it detects constant acceleration and linear, but not quadratic drag. As additional noise is introduced, the models erroneously adopt a term proportional to

ball height. It should be noted that, in these simulations, the factor multiplying v^2 in S3, namely $\frac{1}{2m}\rho A C_D$, does not exceed 0.08, except very early in the balls' trajectories when v is small and v^2 even smaller. The consequence of this observation is that even if this factor were constant with respect to velocity, SINDy and other model discovery methods would have a difficult time accurately detecting it because it is so small relative to the other effects present in the experiment. It should be noted that even if the number of measurements is expanded by increasing the duration of the simulations, SINDy tends to adjust the constant acceleration and linear drag coefficients to match the data rather than adopting a quadratic drag term. Figure S7 demonstrates just how closely linear drag can mimic quadratic drag as a ball approaches terminal velocity. If the amount of data is increased by instead collecting more measurements over a shorter time span[3] we saw no improvements in the ability of the model to detect a quadratic drag term. Similarly, SINDy accounted for increases in the density of the fluid through which the balls fall by adjusting the gravitational constant and linear drag term.

REFERENCES

Mangan NM, Brunton SL, Proctor JL, Kutz JN. Inferring biological networks by sparse identification of nonlinear dynamics. *IEEE Transactions on Molecular, Biological, and Multi-Scale Communications* **2** (2016) 52–63.

Brown PP, Lawler DF. Sphere drag and settling velocity revisited. *Journal of environmental engineering* **129** (2003) 222–231.

[3] We experimented with increasing the sampling rate to 60 measurements per second over 3.33 seconds.
